# Experimental data from flesh quality assessment and shelf life monitoring of high pressure processed European sea bass (*Dicentrarchus labrax*) fillets

**DOI:** 10.1016/j.dib.2019.104451

**Published:** 2019-08-29

**Authors:** Liliana Anjos, Patricia I.S. Pinto, Theofania Tsironi, George Dimopoulos, Soraia Santos, Cátia Santa, Bruno Manadas, Adelino Canario, Petros Taoukis, Deborah M. Power

**Affiliations:** a Comparative Endocrinology and Integrative Biology Group (CEIB), Centro de Ciencias do Mar (CCMAR), University of Algarve, Faro, Portugal; b Laboratory of Food Chemistry and Technology, School of Chemical Engineering, National Technical University of Athens (NTUA), Greece; c Center for Neuroscience and Cell Biology (CNC), University of Coimbra, Portugal; d Institute for Interdisciplinary Research, University of Coimbra (IIIUC), Portugal

**Keywords:** Colour and texture, European sea bass, Fish fillet, High pressure, 16S rRNA microbiome, Quantitative proteomics

## Abstract

Fresh fish are highly perishable food products and their short shelf-life limits their commercial exploitation and leads to waste, which has a negative impact on aquaculture sustainability. New non-thermal food processing methods, such as high pressure (HP) processing, prolong shelf-life while assuring high food quality. The effect of HP processing (600MPa, 25 °C, 5min) on European sea bass (*Dicentrarchus labrax*) fillet quality and shelf life was investigated. The data presented comprises microbiome and proteome profiles of control and HP-processed sea bass fillets from 1 to 67 days of isothermal storage at 2 °C. Bacterial diversity was analysed by Illumina high-throughput sequencing of the *16S rRNA* gene in pooled DNAs from control or HP-processed fillets after 1, 11 or 67 days and the raw reads were deposited in the NCBI-SRA database with accession number PRJNA517618. Yeast and fungi diversity were analysed by high-throughput sequencing of the internal transcribed spacer (ITS) region for control and HP-processed fillets at the end of storage (11 or 67 days, respectively) and have the SRA accession number PRJNA517779. Quantitative label-free proteomics profiles were analysed by SWATH-MS (Sequential Windowed data independent Acquisition of the Total High-resolution-Mass Spectra) in myofibrillar or sarcoplasmic enriched protein extracts pooled for control or HP-processed fillets after 1, 11 and 67 days of storage. Proteome data was deposited in the ProteomeXchange Consortium via the PRIDE partner repository with the dataset identifiers PXD012737. These data support the findings reported in the associated manuscript “High pressure processing of European sea bass (*Dicentrarchus labrax*) fillets and tools for flesh quality and shelf life monitoring”, Tsironi et al., 2019, JFE 262:83–91, doi.org/10.1016/j.jfoodeng.2019.05.010.

**Table:** Specifications Table

Subject area	*Food science*
More specific subject area	*Food processing, Aquaculture*
Type of data	*Tables, figures, supplementary tables*
How data was acquired	*CR-Minolta chromameter, Stable Micro Systems TA-XT2i texture analyzer, Illumina MiSeq, ABSciex Triple TOF* 5600 LC*-SWATH-MS system with information-dependent acquisition (IDA)*
Data format	*Raw, metadata*
Experimental factors	*Marine cultured sea bass were refrigerated on ice, industrially scaled, the head removed, fish filleted and submitted to high pressure or not (control) and then isothermally stored (2°C) for different periods.*
Experimental features	*Colour and texture measurements; DNA extraction, quality evaluation, microbiome library construction of 16S rRNA and internal transcribed spacer PCR amplified regions, sequencing and bioinformatics analysis; protein extractions, quality and quantity estimation, LC-SWATH-MS identification and quantification.*
Data source location	*Koropi, Attica, Greece (37°53′12.4″N; 23°52′40.4″E)*
Data accessibility	*Data is available in this article and at**https://www.ncbi.nlm.nih.gov/sra/**(projects PRJNA517618 and PRJNA517779) and at the ProteomeXchange Consortium* via *the PRIDE repository with identifier PXD012737.*
Related research article	Theofania Tsironi, Liliana Anjos, Patricia I. S. Pinto, George Dimopoulos, Soraia Santos, Cátia Santa, Bruno Manadas, Adelino Canario, Petros Taoukis, Deborah Power *“High pressure processing of European sea bass (Dicentrarchus labrax) fillets and tools for flesh quality and shelf life monitoring”*. *Journal of Food Engineering* 262, 83–91 https://doi.org/10.1016/j.jfoodeng.2019.05.010

**Table:** 

**Value of the Data** • The data represents an integrated view of the impact of novel non-thermal processing technologies on fish fillet quality and safety and makes a relevant contribution to the food loss and waste paradigm. • A metagenomics approach for the microbiome used *16s rRNA* (bacteria) and *internal transcribed spacer* (for yeast and fungi) sequencing to provide a global and in-depth overview of microorganism diversity and dynamics in control and HP processed fillets during storage at 2 °C. • SWATH-MS proteomics is used to give insight for the first time into the global impact of HP processing and cold storage on the proteins in sea bass fillets. • The study demonstrates how integration of conventional experimental methods and novel omics technologies corroborate and substantially extend the outcome of previous work. • HP processing is revealed as an emerging processing approach that prolongs the shelf life of highly perishable commodities such as fish fillets.

## 1 Data

**Fig. 1:**
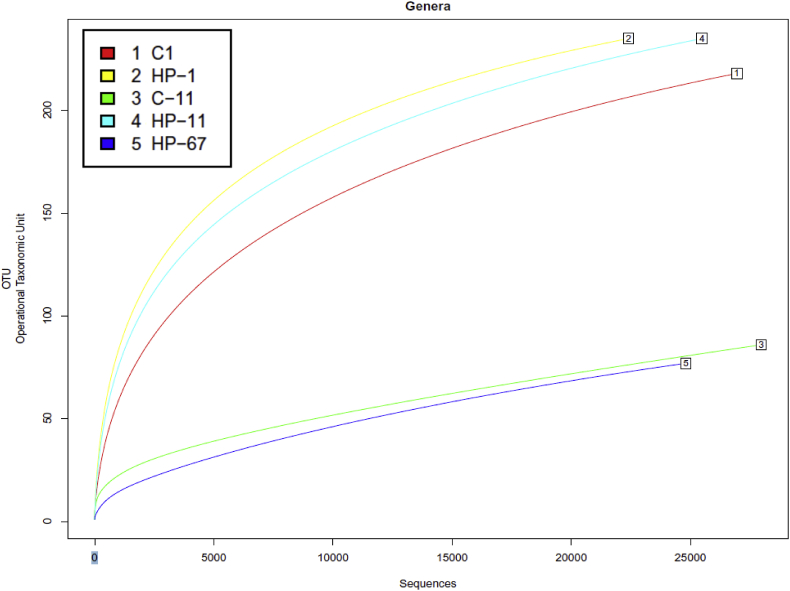
Rarefaction plots for the microbiome libraries sequenced by 16S rRNA gene sequencing (bacterial diversity), determined based on all operational taxonomic units (OTUs) found at the genus level, including annotated and non-annotated sequences (no hits).

**Fig. 2:**
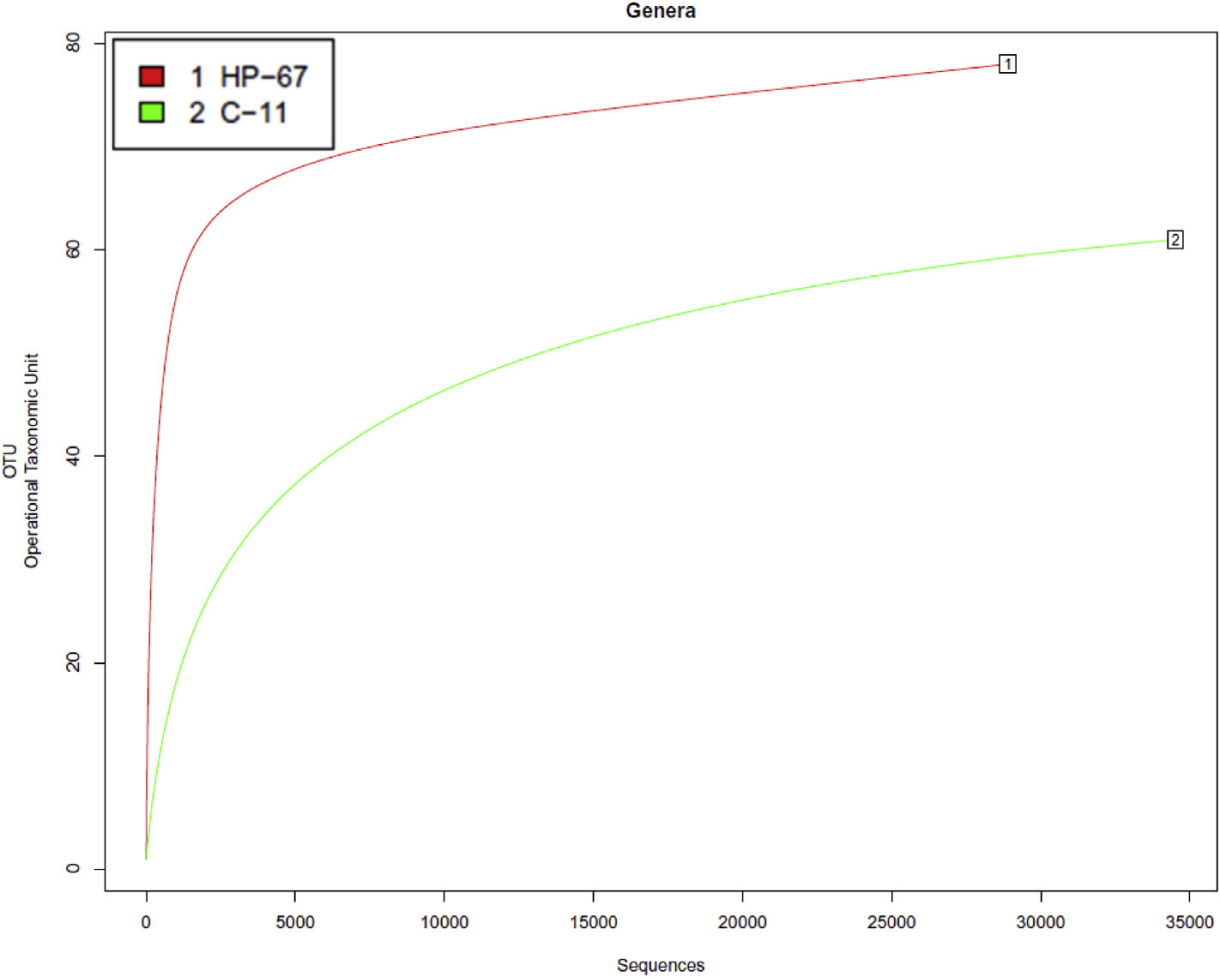
Rarefaction plots for the microbiome libraries directed at the fungal ITS region, determined based on all operational taxonomic units (OTUs) found at the genus level, including annotated and non-annotated sequences (no hits).

**Fig. 3:**
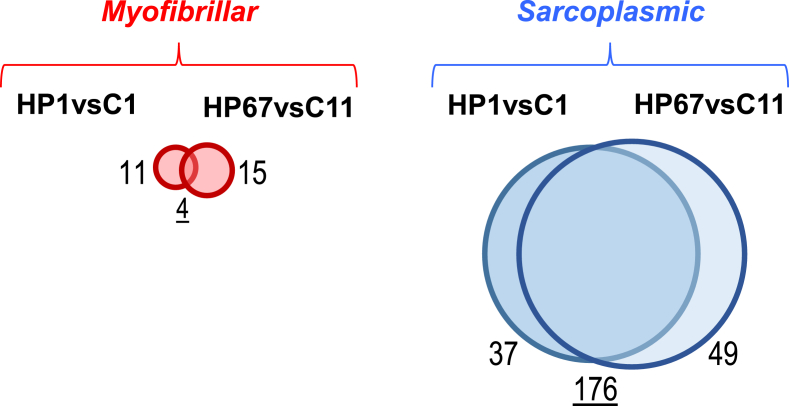
Venn diagrams representing the number of proteins that had a more than 2-fold modified abundance in HPP sea bass fillets compared to the control groups (C) at the equivalent storage time in days (eg. short-term, C1 and HP1 or long-term, C11, HP67). Proteins were identified and quantified by SWATH proteomics. For Myofibrillar (red) or sarcoplasmic (blue) protein extracts, the size of the circles is proportional to the number of proteins that had a modified expression (the number of proteins are indicated next to the circles). The proteins that changed in the same way between the different treatment groups at the beginning and end of storage are located in the intersection of two circles in the Venn diagram (the number of common proteins is underlined).

Supplementary Table 1 summarizes *16S rRNA* sequencing quality statistics of HP-treated and control fillets at different time-points during storage, and Fig. 1 presents the alpha rarefaction analysis of the same data, showing that all sequencing libraries were near to saturation. Supplementary Table S2 lists the main bacterial genera detected and their relative abundance in each sequencing library. Supplementary Table S3 lists all bacterial genera detected including those present in low abundance (<1% of the total genera detected). Similarly, Supplementary Tables S4 and S5 contain, respectively, lists of the most abundant bacteria species and complete list of detected bacteria, together with the Shannon diversity index and CHAO1 Richness Estimation for each library. Supplementary Table S6 summarizes the ITS sequence quality statistics for yeast and fungi of HP-treated and control fillets at the end of storage and Fig. 2 presents the alpha rarefaction analysis of the same data, showing that all sequencing libraries were near to saturation. Supplementary Tables S7 and S9 list, respectively, the main fungal genera and species detected by ITS in 1% or more of the reads. Supplementary Tables S8 and S10 list, respectively, the full list of fungal genera and species detected by ITS. Supplementary Table S11 summarizes the number of proteins and peptides from myofibrillar and sarcoplasmic-enriched protein extracts that were identified or quantified by SWATH analysis in HP-processed or control fillets at different storage times. Supplementary Tables S12 and S13 (spreadsheet format) list the quantification parameters of the total proteome profiles of myofibrillar and sarcoplasmic extracts, respectively. Supplementary Table S14 lists the 38 proteins from the myofibrillar extract and Supplementary Table S15 lists the 263 proteins from the sarcoplasmic extract (in spreadsheet format) that changed greater than 2-fold between the control and HP-processed fillets and/or between cold storage times; the relative proportions of the proteins are summarized in Fig. 3.

## 2 Experimental design, materials, and methods

### 2.1 Experimental set-up and fish fillet samples

European sea bass (*Dicentrarchus labrax*) (weight: 110 ± 10 g) from Nireus Aquaculture, were stunned on ice slush (0 °C) immediately upon harvesting, size sorted and transported to the filleting line within 1 day. In the industrial facilities the fish was filleted after descaling and discarding of the head. Sea bass fillets were rinsed in tap water, placed in polystyrene boxes with appropriate quantities of flaked ice (0 °C) and transported to the Laboratory of Food Chemistry and Technology (NTUA, Athens, Greece) within 2–3 hours.

A laboratory pilot scale Food Pressure Unit (FPU 1.01, Resato International BV, Roden, Holland) with a maximum operating pressure of 1000 MPa was used for high pressure treatments. The high-pressure unit had a 1.5 L volume and a multivessel system consisting of six vessels of 45 mL capacity each. All high-pressure vessels were surrounded by a circulating jacket connected to a heating-cooling system. The pressure transmitting fluid was polyglycol ISO viscosity class VG 15 (Resato International, BV, Roden, Holland). Fish fillets were packed in pouches (two per pouch) consisting of a multilayer (PP-PE) packaging material. HP processed fillets were vacuum-packed and treated in-pack at 600 MPa and 25 °C for 5 min, according to Ref. [1]. The non-processed (control) samples were stored aerobically in non-sealed pouches, simulating conventional aerobic retail display facilities.

Control and HP-treated (HP) fillets were stored at 2 °C under controlled isothermal conditions in high-precision (±0.2 °C) low-temperature incubators (Sanyo MIR 153, Sanyo Electric, Ora-Gun, Gunma, Japan), monitored with electronic, programmable miniature data-loggers (COX TRACER ®, Belmont, NC). Samples of ca 5 cm^2^ sections were collected from fillets at different storage times: 1 day (C1 and HP1 groups), 11 days (C11 and HP11), 32 days (HP32) and 67 days (HP67). Samples from control fillets were not collected for the last two time points since they had deteriorated to unacceptable levels after 11 days of storage.

### 2.2 Colour and texture parameters

Fillet colour change over time and treatment was measured using a CR-Minolta Chromameter (Minolta Co., Chuo-Ku, Osaka, Japan) with an 8mm diameter measuring area. The instrument was standardized under “C” illuminant conditions according to the CIE (Commission International de l’ Eclairage) using a standard white reference tile (calibration plate CR-200, L = 97.50, a = −0.31, b = −3.83). Measurements of CIELab values (L-value: lightness, a-value: redness and greenness, b-value: yellowness and blueness) were made in three different points of the flesh on the upper side of the fillet (lateral line) and 3 fillets were tested per group and sampling point.

Texture parameters were evaluated using a texture analyser with a load cell of 5 kg (TA-XT2i, Stable Micro Systems, Godalming, Surrey, United Kingdom). A compression aluminium plate of 75 mm or 20 mm diameter was selected. Double compression was applied to construct the texture profile analysis (TPA) parameters of 3 different fillets per group and sampling point. The aluminium plate approached the sample at the speed of 0.5 mm/sec and pressed 2 mm into the fish flesh. Then the force was reduced and the sample was allowed to rebound 5 s before the second compression. Force-time curves were obtained and texture parameters (hardness, cohesiveness and adhesiveness) were determined using the Texture Expert Exceed Application (Version 2.64, Stable Micro Systems Ltd) [2].

### 2.3 DNA extracts

Total DNA was separately extracted from 4 fillets from each of the groups C1, C11, HP1, HP11 and HP67, using a DNeasy Blood & Tissue Kit (Qiagen) with modifications. The excision of approx. 40 mg taken from the fillet surface was carried out under sterile conditions. Muscle samples were cut into small pieces, added to 2 ml sterile tubes with 200 μl of lysis buffer (20Mm Tris-HCl, pH 8; 2mM sodium EDTA; 1.2% Triton X-100; 40 mg/ml Lysozyme), 200 μl of AL buffer and approx. 400 mg of glass beads (0.1mm zirconia/silica beads, Biospec). The tubes were maintained on ice until mechanical disruption was carried out at room temperature in a Bertin Precellys 24 homogeniser; the disruption procedure was repeated in two pulses of 15 s at 6800 rpm. Tubes were incubated for 30 min at 37 °C to complete the enzymatic lysis, and then 25 μl of proteinase K (supplied with the kit) was added to each tube, which were incubated for a further 30 min at 56 °C. Tubes were centrifuged for 1 min at 8000 rpm and the lysed supernatant was recovered and incubated with RNAse A (10 μl of 10 mg/ml) for 10 min at room temperature. The remaining steps of the extraction method were performed as recommended in the protocol of the DNeasy Blood & Tissue Kit, with a final elution of extracted DNA in 50 μl of EB buffer (10Mm Tris-HCl, pH 8). DNA quality and integrity were analysed using a Nanodrop spectrophotometer and 1% agarose gel electrophoresis.

### 2.4 Microbiome library preparation, sequencing and bioinformatics analyses

DNA pools were prepared from individual DNA extracts (ca. 250 ng each) and then concentrated with an RNeasy Micro Kit (Qiagen), following the manufacturer's instructions, and eluted into 22 μl EB buffer. Five bacterial microbiome libraries (groups C1, C11, HP1, HP11 and HP67) were prepared from 75 to 210 ng of pooled DNA using the 16S Metagenomic Sequencing Library Preparation protocol (for the Illumina MiSeq system) and primers spanning the V3 and V4 regions of the 16S rRNA gene [3]. Two fungal microbiome libraries (C11 and HP67) were prepared from 30 to 130 ng of pooled DNA, using the same protocol as outlined above but with a set of primers directed against regions ITS3-ITS4 from *Ascomycetes* and *Basidiomycetes* [4]. Libraries were sequenced by Lifesequencing S.L.-ADM (Valencia, Spain) using an Illumina MiSeq instrument. Sequence data analysis, included: trimming of adaptors and filtering of low-quality reads that had a minimum value Q20 and minimum read length of 200 nucleotides; removal of chimeras and identification and classification of operational taxonomic units (OTU). Data analysis was carried out as described in Ref. [5]. For OTU assignment, sequence similarity searches were made against the NCBI 16S rRNA database (for the 16S libraries) or against an ITS built database of fungal ITS sequences extracted from NCBI (for the ITS libraries), with a cut-off set at 97% identity. Global microbiome data analyses were performed as described in Ref. [5], using a pipeline developed by Lifesequencing S.L.-ADM, and CD-HIT software [6] was used for hierarchy clustering.

### 2.5 Quantitative proteomics

Differences in the protein content of control (C1, C11) and HP fillets (HP1, HP67) were analysed by SWATH-MS (Sequential Window data independent Acquisition of the Total High-resolution-Mass Spectra) using pools of myofibrillar or sarcoplasmic enriched protein extracts (n = 6 per group). Myofibrillar and sarcoplasmic enriched protein extracts (based on [7] with modifications) were prepared from individual fillets (n = 6 individuals per group). The extraction buffer used for the myofibrillar extracts was 50 mM Tris buffer pH 7.2, 100 mM DTT, 1.7% SDS and for sarcoplasmic extracts was 10 mM Tris buffer pH 7.2 and both buffers contained a protease inhibitor cocktail (Sigma, US). The muscle sample (0.5 g) was reduced to a powder in liquid nitrogen and ≈0.1 g was solubilized in extraction buffer (ratio: 1 g of tissue/8 ml of buffer), and mechanically disrupted using 0.5 mm zirconia/silica beads (Biospec) in a Bertin Precellys 24 homogeniser using 2 cycles of 20 s at 6800 rpm. The homogenates were boiled at 95 °C for 10 min and allowed to cool at room temperature. The soluble protein fraction was separated by centrifugation at 28,000 *g* for 15 min at 20 °C, alkylated with 40% acrylamide solution (1:15 of acrylamide: protein solution v/v) and stored at −80 °C. The soluble protein concentration was determined using the Bradford assay with a bovine serum albumin standard set (#500–0006 and #500–0207, BioRad, USA). The electrophoretic profile of the myofibrillar and sarcoplasmic enriched protein extracts was analysed by 1D SDS-PAGE (12%) according to Ref. [8] followed by Coomassie blue staining.

For SWATH-MS proteomic analysis the protein extracts were gel digested using the short-GeLC method [9]. Briefly, a volume corresponding to 50 μg was prepared by pooling protein extracts from 6 individuals/treatment, and a further sample was prepared that consisted of 10 μg from each experimental group pool. The sample pools were fractionated by SDS- PAGE. Selected gel regions were excised and processed as in Ref. [10], for destaining, dehydration, rehydration, in-gel protein digestion using trypsin, peptide extraction and solid phase extraction with C18 sorbent (OMIX tip, Agilent Technologies). Samples were analysed using SWATH-MS on a Triple TOF™ 5600 System (Sciex®, Framingham, MA) using information-dependent acquisition (IDA) of the pooled mixture of all samples; followed by SWATH acquisition of each individual group pool. Peptide separation was performed using liquid chromatography (nanoLC Ultra 2D, Eksigent) on a ChromXP C18CL reverse phase column (300 μm × 15 cm, 3 μm, 120 Å, Eksigent) at 5 μL/min with a 45 min gradient from 2% to 35% acetonitrile in 0.1% FA with 5% DMSO, and the peptides were eluted into the mass spectrometer using an electrospray ionization source (DuoSpray™ Source, Sciex).

IDA experiments were performed by analysing each fraction of the pooled mixture. The mass spectrometer was set for IDA scanning full spectra (350–1250 m/z) for 250 ms, followed by up to 100 MS/MS scans (100–1500 m/z from a dynamic accumulation time – minimum 30 ms for precursors above the intensity threshold of 1000 counts per second (cps) – in order to maintain a cycle time of 3.3 s). Candidate ions with a charge state between +2 and + 5 and counts above a minimum threshold of 10 cps were isolated for fragmentation and one MS/MS spectra was collected before adding those ions to the exclusion list for 25 s (mass spectrometer operated by Analyst® TF 1.7, Sciex). Rolling collision energy was used with a collision energy spread (CES) of 5.

The SWATH setup was as in Ref. [11] with the same chromatographic conditions used for IDA acquisitions. Briefly, the mass spectrometer was operated in a looped product ion mode. The SWATH MS setup was designed specifically for the samples to be analysed, in order to adapt the SWATH windows to the complexity of this batch of samples. A set of 60 SWATH windows of variable width (containing 1 m/z for window overlap) was constructed covering the precursor mass range of 350–1250 m/z. A 200 ms survey scan (350–1250 m/z) was acquired at the beginning of each cycle for instrument calibration and SWATH MS/MS spectra were collected from 100 to 1500 m/z for 50 ms resulting in a cycle time of 3.3 s from the precursors ranging from 350 to 1250 m/z. The collision energy for each window was determined according to the calculation for a charge +2 ion centered upon the window with variable CES (Collision Energy Spread) according to the window. Peptide identification and library generation were carried out using Protein Pilot software (v5.0, Sciex®) and the following search parameters: 1) comparison against the predicted proteins from the sea bass genome database (June 2012 draft assembly dicLab v1.0c with annotation from July 2013; file diclab1_pep.faa.gz downloaded from http://seabass.mpipz.mpg.de/DOWNLOADS/ [12]; 2) acrylamide alkylation; 3) trypsin digestion (Paragon™ Algorithm).

The SWATH™ processing plug-in for PeakView™ (v2.0.01, ABSciex®) was used for SWATH data processing, as described in Ref. [9] with minor modifications. Peptides were selected automatically from the library following the criteria: i) unique peptides for a specific targeted protein were ranked by the intensity of the precursor ion from the IDA analysis; ii) peptides that contained biological modifications and/or were shared between different protein entries/isoforms were excluded. Up to 15 peptides were chosen per protein, and SWATH™ quantification was attempted for all proteins considered as positive identifications. Peptide retention time was adjusted using the malE-GFP peptides. Up to 5 target fragment ions per peptide were automatically selected and the peak groups were scored following the criteria described in Ref. [13]. Protein levels were estimated by summing all the transitions from all the peptides for a given protein as described in Ref. [14] and normalized to the total intensity of the sample at the protein level. The mass spectrometry proteomics data have been deposited with the ProteomeXchange Consortium [15] via the PRIDE [16] partner repository with the dataset identifier PXD012737. In proteomic data analysis, the count ratio method was used to compare the relative protein abundance modified by HP (HP1/C1; HP67/C11) or storage time (HP1/HP67; C1/C11) in each type of extract, considering a 2-fold change as the threshold.

Protein lists that changed with the experimental conditions were analysed using the proportional Venn diagram tool BioVenn (http://www.biovenn.nl) [17], UNIPROT (https://www.uniprot.org/) [18] and BRENDA (https://www.brenda-enzymes.org) [19] for screening of enzymes, structural proteins and other classifications. The zebrafish (*Danio rerio*) orthologues for the sea bass proteins were obtained using stand-alone BlastX (with an E value < 10^−10^) against the Ensembl zebrafish protein predictions (GRC Zebrafish Build 10, INSDC Assembly GCA_000002035.3 from Sep 2014, https://www.ensembl.org [20]) in order to establish a comparative annotation.

## Authors contributions

TT, PT and DMP conceived the study; AVMC, PT and DMP provided the resources; TT and GD performed the High-Pressure experiments, fish sampling and conventional microbiological, texture and colour analysis; Microbiome analysis of the fish fillets was performed by PISP and SS. LA, CS and BM carried out the protein analysis and SWATH-proteomics and data analysis; TT, PT, DMP, AVMC, LA, PISP critically analysed and reviewed all the results of the study; TT, LA, PISP and DMP drafted the manuscript, which was critically reviewed and approved by all authors.

## References

[1] Tsironi T., Maltezou I., Tsevdou M., Katsaros G., Taoukis P. (2015). High-pressure cold pasteurization of gilthead seabream fillets: selection of process conditions and validation of shelf life extension. Food Bioprocess Technol..

[2] Sigurgisladottir S., Hafsteinsson H., Jonsson A., Lie Ø., Nortvedt R., Thomassen M., Torrissen O. (1999). Textural properties of raw salmon fillets as related to sampling method. J. Food Sci..

[3] Klindworth A., Pruesse E., Schweer T., Peplies J., Quast C., Horn M., Glöckner F.O. (2013). Evaluation of general 16S ribosomal RNA gene PCR primers for classical and next-generation sequencing-based diversity studies. Nucleic Acids Res..

[4] Toju H., Tanabe A.S., Yamamoto S., Sato H. (2012). High-coverage ITS primers for the DNA-based identification of ascomycetes and basidiomycetes in environmental samples. PLoS One.

[5] Codoñer F.M., Ramirez-Bosca A., Climent E., Carrion-Gutierrez M., Guerrero M., Perez-Orquin J.M., Horga de la Parte J., Genoves S., Ramon D., Navarro-Lopez V., Chenoll E. (2018). Gut microbial composition in patients with psoriasis. Sci. Rep..

[6] Fu L., Niu B., Zhu Z., Wu S., Li W. (2012). CD-Hit: accelerated for clustering the next-generation sequencing data. Bioinformatics.

[7] Pazos M., Méndez L., Vázquez M., Aubourg S.P. (2015). Proteomics analysis in frozen horse mackerel previously high-pressure processed. Food Chem..

[8] Laemmli U.K. (1970). Cleavage of structural proteins during the assembly of the head of bacteriophage T4. Nature.

[9] Anjo S.I., Santa C., Manadas B. (2015). Short GeLC-SWATH: a fast and reliable quantitative approach for proteomic screenings. Proteomics.

[10] Santa C., Anjo S.I., Manadas B. (2016). Protein precipitation of diluted samples in SDS-containing buffer with acetone leads to higher protein recovery and reproducibility in comparison with TCA/acetone approach. Proteomics.

[11] Gillet L.C., Navarro P., Tate S., Röst H., Selevsek N., Reiter L., Bonner R., Aebersold R. (2012). Targeted data extraction of the MS/MS spectra generated by data-independent acquisition: a new concept for consistent and accurate proteome analysis. Mol. Cell. Proteom..

[12] Tine Mbaye, Kuhl Heiner, Gagnaire Pierre-Alexandre, Louro Bruno, Desmarais Erick, Martins Rute S.T., Hecht Jochen, Knaust Florian, Belkhir Khalid, Klages Sven, Dieterich Roland, Stueber Kurt, Piferrer Francesc, Guinand Bruno, Bierne Nicolas, Volckaert Filip A.M., Bargelloni Luca, Power Deborah M., Bonhomme François, Canario Adelino V.M., Reinhardt Richard (2014). European sea bass genome and its variation provide insights into adaptation to euryhalinity and speciation. Nat. Commun..

[13] Lambert J.P., Ivosev G., Couzens A.L., Larsen B., Taipale M., Lin Z.Y., Zhong Q., Lindquist S., Vidal M., Aebersold R. (2013). Mapping differential interactomes by affinity purification coupled with data-independent mass spectrometry acquisition. Nat. Methods.

[14] Collins B.C., Gillet L.C., Rosenberger G., Röst H.L., Vichalkovski A., Gstaiger M., Aebersold R. (2013). Quantifying protein interaction dynamics by SWATH mass spectrometry: application to the 14-3-3 system. Nat. Methods.

[15] Deutsch E.W., Csordas A., Sun Z., Jarnuczak A., Perez-Riverol Y., Ternent T., Campbell D.S., Bernal-Llinares M., Okuda S., Kawano S., Moritz R.L., Carver J.J., Wang M., Ishihama Y., Bandeira N., Hermjakob H., Vizcaino J.A. (2017). The ProteomeXchange consortium in 2017: supporting the cultural change in proteomics public data deposition. Nucleic Acids Res..

[16] Perez-Riverol Y., Csordas A., Bai J., Bernal-Llinares M., Hewapathirana S., Kundu D.J., Inuganti A., Griss J., Mayer G., Eisenacher M., Perez E., Uszkoreit J., Pfeuffer J., Sachsenberg T., Yilmaz S., Tiwary S., Cox J., Audain E., Walzer M., Jarnuczak A.F., Ternent T., Brazma A., Vizcaino J.A. (2019). The PRIDE database and related tools and resources in 2019: improving support for quantification data. Nucleic Acids Res..

[17] Hulsen T., de Vlieg J., Alkema W. (2008). BioVenn - a web application for the comparison and visualization of biological lists using area-proportional Venn diagrams. BMC Genomics.

[18] The UniProt Consortium (2017). UniProt: the universal protein knowledgebase. Nucleic Acids Res..

[19] Placzek S., Schomburg I., Chang A., Jeske L., Ulbrich M., Tillack J., Schomburg D. (2017). BRENDA in 2017: new perspectives and new tools in BRENDA. Nucleic Acids Res..

[20] Aken B.L., Achuthan P., Akanni W., Amode M.R., Bernsdorff F., Bhai J., Billis K., Carvalho-Silva D., Cummins C., Clapham P. (2016). Ensembl 2017. Nucleic Acids Res..

